# Borderline paratubal cyst: a case report

**Published:** 2012-11-16

**Authors:** Fatima Zohra Fdili Alaoui, Hinde El Fatemi, Hekmat Chaara, Molay Abdilah Melhouf, Afaf Amarti

**Affiliations:** 1 Department of obstetrics and gynecology II, University Hospital Hassan II Fez, University Sidi Mohamed Ben Abdellah, Morocco; 2 Department of anatomy, university Hospital Hassan II Fez, University Sidi Mohamed Ben Abdelah, Morocco

**Keywords:** Paratubal cyst, borderline, torsion, treatment

## Abstract

Borderline para-ovarian cysts (PCs) are rare entities. They are commonly present in the third decade. Borderline PCs are often discovered fortuitously on routine ultrasound examination or they are common incidental findings during a laparotomy. They must be differentiated from simple ovarian cysts, peritoneal inclusion cysts and hydrosalpinges on ultrasound sonography. Papillary projections on the cyst wall should be searched carefully to suggest diagnosis. The treatment is surgical including fertility -sparing operation or more radical surgery depending on the case. The prognosis is good because borderline PCs are usually early-stage at diagnosis. Here is a report of a 38- year old woman with a borderline paratubal cyst. Adnexal torsion of hydrosalpinx was suspected; thus, she underwent an urgent surgery. Cystectomy was performed without rupture. The final diagnosis revealed a borderline PC. The patient underwent a radical surgery. Currently, she has had no evidence of disease recurrence.

## Introduction

Paraovarian or paratubal cysts constitue about 10-20% of adnexial masses. They are usually asymptomatic and benign [1, 2]. Borderline paratubal cysts are identified as epithelial proliferation without stromal invasion; these rare tumors have been reported only as case reports in the literature. We report a case of a 38 - year woman with a borderline paratubal cyst. She underwent an urgent surgery since we suspected an adnexal torsion of hydrosalpinx.

## Patient and observation

**Figure 1:**
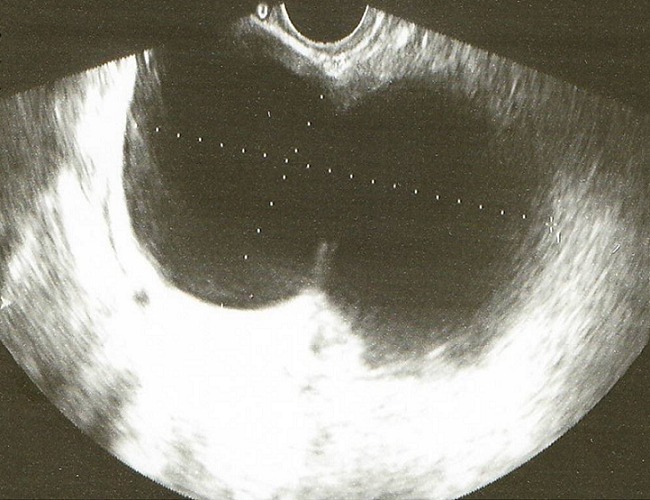
The sonographic evaluation showed a 100/52mm right oblong adnexal cyst suggesting a hydrosalpinx

**Figure 2:**
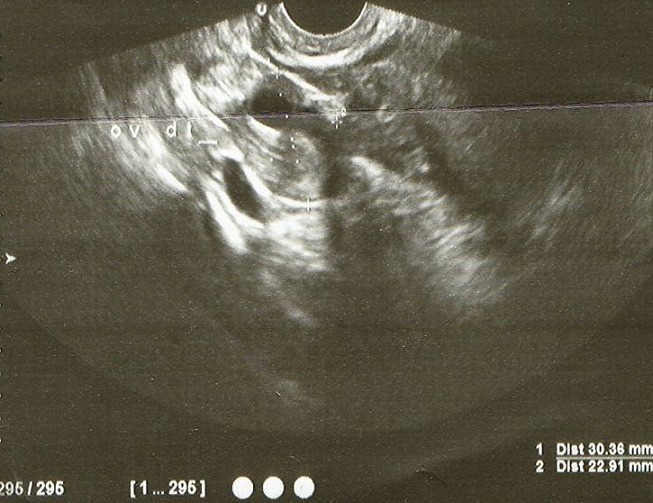
Ultrasound showed that the cyst is clearly separated from the ipsilateral ovary

**Figure 3:**
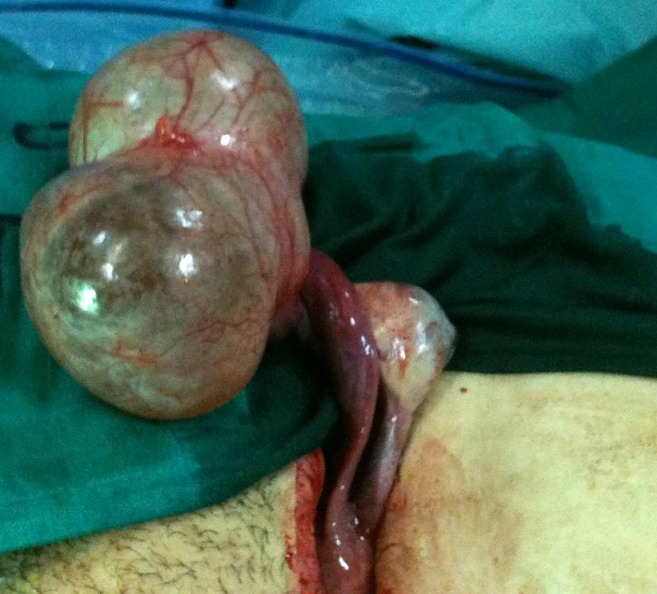
At surgery, a 10cm twisted right paratubal cyst were diagnosed

**Figure 4:**
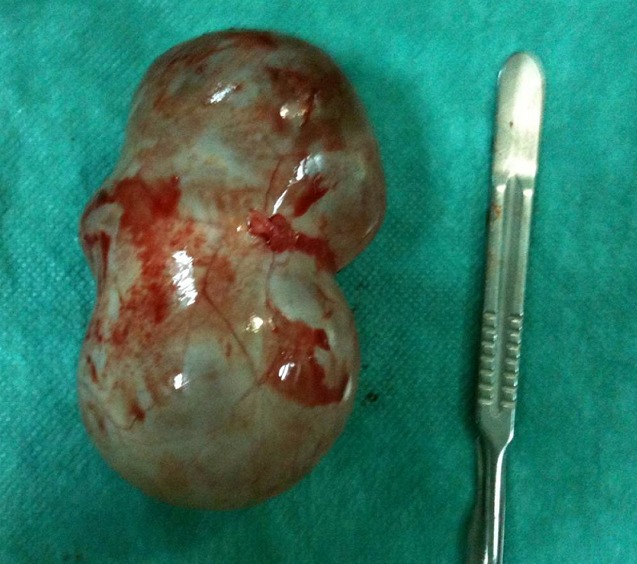
Cystectomy was performed without rupture

A 38-year old woman, mother of three children was referred to our gynecological emergencies with an acute onset of persistent sharp right lower quadrant pain as well as nausea and vomiting. Her gynecological exam findings were right lower quadrant pain and rebound tenderness with right lateral uterine wheelbase. Sonographic evaluation revealed a 100/52mm right oblong adnexal cyst, clearly separated from the ipsilateral ovary suggesting a hydrosalpinx (Figure 1, Figure 2). The patient underwent laparotomy due to a torsion indicated by the clinical and paraclinical examination. We opted for laparotomy for two reasons.First, the patient had a triple cicatricial uterus. Second, laparoscopy colomn is not available at the emergencies. During the surgery, a 10cm twisted right paratubal cyst and normal bilateral ovaries were diagnosed (Figure 3). Cystectomy was performed without rupture (Figure 4) and the cyst was sent to pathology. Grossly, the paraovarian cyst measured 10X5cm, contained serous fluid, rare papillary projections measuring 0,5cm were noted in the outer surface of the cyst.

**Figure 5:**
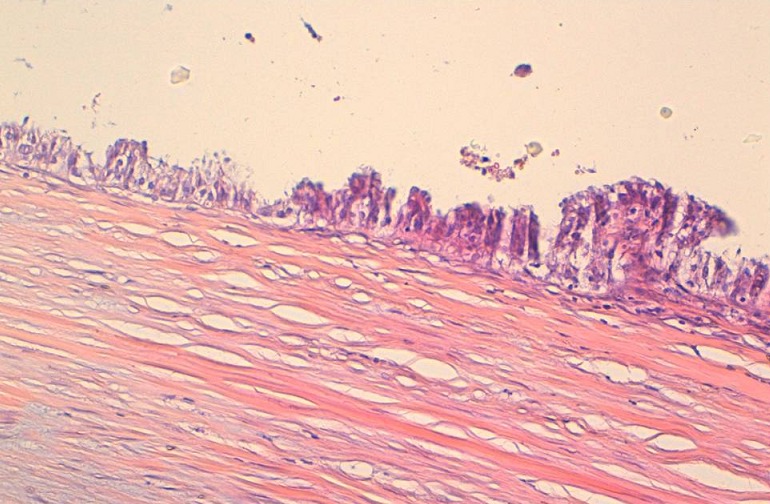
Borderline paratubal cyst: The epithelium showed nuclear stratification, gland formation and atypical papillary proliferations. No invasive features were recognized

Histologically, papillary epithelial proliferation was recognized. The epithelium showed nuclear stratification, gland formation and atypical papillary proliferations. No invasive features were recognized. The tumor was pathologically diagnosed as borderline serous papillary tumor within PC (Figure 5). Abdominal pelvic CT scan and tumor markers (CA125) were normal. The patient underwent radical surgery: washings, hysterectomy, bilateral salpingo-oophorectomy, omentectomy, biopsies. During the final pathology review, no metastatic disease was observed. After one year of follow up, the patient was free of disease recurrence.

## Discussion

Paraovarian cysts (PCs) account for about 10-20% for all adnexal masses [1, 2]. They may be wolffian duct or paramesonephrotic duct remains, they arise from the broad ligament between the fallopian tube and the ovary [3].

Although Paraovarian cysts are common disorders, borderline PCs are rare, they have been reported only as case reports in the literature. The average age noted by most authors is 31years [4]. Borderline PCs are often discovered fortuitously on routine ultrasound examination or they are common incidental findings during laparotomy, however sometimes they become symptomatic with acute abdominal pain when they are torsed or ruptured [5, 6]. Transvaginal sonography(TVS) may raise some signs that enable a correct differential diagnosis. It also has the advantage of allowing a dynamic evaluation of the patient in comparaison with any other imaging modality (computed tomography, magnetic resonance imaging). The differential diagnosis includes a simple ovarian cyst, peritoneal inclusion cyst and hydrosalpinx; In fact, the proximity to the ovary, the possible presence of septations and small parietal papillae, should be considered in the differencial diagnosis along with peritoneal inclusion cyst and hydrosalpinx (Table 1). Peritoneal inclusion cysts are multilocular cystic masses with an irregular, star like morphology and no proper wall; septations are multiple and free to oscillate when moving the probe (flapping sail sign). Hydrosalpinges are tortuous convoluted cystic adnexal masses delimited by a distinct wall and showing small hyperechoic mural nodules on the cross-section of the salpinx, named (beads-on -a-string). Both hydrosalpinges and paraovarian cysts have the (split sign) identified by pushing the tip of the vaginal probe between this structures and ipsilateral ovary [7].

**Table 1:** Differential diagnosis of paraovarian cysts, hydrosalpinges and peritoneal inclusion cysts

Diagnosis	Paraovarian cysts	Hydrosalpinges	Peritoneal Inclusion cysts
Ipsilateral ovary	Yes	Yes	Yes
Morphology	Ovoid	Tubular	Irregular
Proper wall	Yes	Yes	No
Papillae	Yes	No	Yes
Beads -on- a -string	No	Yes	No
Complete septa	Rare	No	Frequent
Incomplete septa	No	Frequent	Rare
Flapping sail sign	No	No	Yes
Split sign	Yes	Yes	No

Although little is known regarding the findings of preoperative imaging studies of patients presenting borderline paraovarian cysts, the authors described low-level echoes seen within the cyst and papillary projections on the cyst wall which should be searched carefully [8]. However, paraovarian cysts are sometimes removed, and are considered as benign; it is not until pathological assessment that the borderline tumor is recognized. This is exactly what happened in our case.

When ultrasound shows papillary projections in the cyst, frozen section analysis must be performed. Unfortunately, in some cases, the absence of typical ultrasound features leads to misdiagnosing these cysts as benign lesions. Borderline paraovarian cysts are usually early-stage at diagnosis. It has been suggested that the histologic appearance of those tumors is identical with that of borderline ovarian tumors; however, it is not known whether their biological behavior is also similar [5]. The intraoperative management includes salpingostomy with tubal cystectomy, partial or complete salpingectomy, hysterectomy and bilateral oophorosalpingectomy, or salpingectomy along with pelvic-aortic lymphadenectomy or pelvic nodal sampling and omentectomy or biopsy with pelvic nodal sampling [9]. In review of the literature, no positive lymph nodes or metastatic disease were found in patients undergoing more comprehensive staging and no recurrence was observed in the follow-up of these patients. [10]. While the optimal procedure is unknown, patients desiring future childbearing may be applying for fertility -sparing operation.If patients have no desire for fertility, more radical surgery may be preferred (washings, hysterectomy, bilateral salpingo-oophorectomy, pelvic-aortic lymphadenectomy, omentectomy, biopsies) [11]. Pelvic aortic lymphadenectomy is still a controversial subject [12]: in our case the treatment was: washings, hysterectomy, bilateral salpingo-oophorectomy, omentectomy and biopsies without pelvic aortic lymphadenectomy. However, close follow-up is needed to detect recurrent disease after conservative, fertility-sparing surgery. Combining routine ultrasonography and markers (CA125 in serous tumor and 19.9 in mucinous tumor) during follow-up examinations and prolongation of such follow-ups after 10 years were suggested [13]. Some cases of relapse have been observed but this had no impact on survival [14].

## Conclusion

Paraovarian cysts are usually asymptomatic and benign. They can become symptomatic when they are torsed or ruptured. Paraovarian cysts are very difficult to diagnose with sonography: The differential diagnosis includes a simple ovarian cyst, peritoneal inclusion cyst and hydrosalpinx. Papillary projections on the cyst wall should be searched carefully to suggest borderline PC diagnosis which still a very rare entity. The treatment included fertility -sparing operation or more radical surgery depending on whether the patient wishes future childbearing or not. Borderline paraovarian cysts are usually early-stage at diagnosis; thus the prognosis still good. Continued reporting of these tumors is essential to understanding the diagnosis and management of this rare neoplasm.
